# Parliament and the Professions

**Published:** 1921-05-28

**Authors:** 


### Parliament and the Professions.

### Arseno-Benzol Treatment.

Mr. R. Richardson asked the Minister of Health as to causes of death through arseno-benzol treatment of venereal diseases. Sir Alfred Mond replied that twenty-one deaths have been reported to his Department, as following the administration of arseno-benzol compounds in treatment centres of venereal diseases. In three of these cases death was not due to the administration of the drug, and in five others it was doubtful how far the drug contributed to the fatal result. The statistics indicate that one death in approximately every 4,300 patients treated for syphilis may count the administration of the drug as a contributing cause, and one in approximately every 30,000 injections.

### Dentists Bill.

The Dentists Bill was read a second time on May 11, and committed to a Standing Committee.

